# A simple method for diagnosing gallbladder malignant tumors with subserosa invasion by endoscopic ultrasonography

**DOI:** 10.1186/s12885-021-08017-x

**Published:** 2021-03-17

**Authors:** Mitsuru Sugimoto, Hiroki Irie, Mika Takasumi, Minami Hashimoto, Yuka Oka, Tadayuki Takagi, Rei Suzuki, Naoki Konno, Hiroyuki Asama, Yuki Sato, Jun Nakamura, Tsunetaka Kato, Ryoichiro Kobashi, Yuko Hashimoto, Shigeru Marubashi, Takuto Hikichi, Hiromasa Ohira

**Affiliations:** 1 Department of Gastroenterology, School of Medicine, Fukushima Medical University, Fukushima, Japan; 2 Department of Endoscopy, Fukushima Medical University Hospital, Fukushima, Japan; 3 Department of Diagnostic Pathology, School of Medicine, Fukushima Medical University, Fukushima, Japan; 4 Department of Hepato-Biliary-Pancreatic and Transplant Surgery, School of Medicine, Fukushima Medical University, Fukushima, Japan

**Keywords:** EUS, Gallbladder malignant tumor, Depth, Subserosa invasion

## Abstract

**Background:**

If the depth of gallbladder malignant tumor (GBMT) invasion is deeper than the subserosa (ss), cholecystectomy is insufficient. In past reports that used endoscopic ultrasonography (EUS) to diagnose the depth of tumor invasion, it was difficult to diagnose GMBT invasion in the ss without a narrow or disrupted lateral hyperechoic layer (LHEL). Therefore, we developed a simple preoperative method to diagnose GBMTs with ss invasion.

**Methods:**

Forty-nine GBMT patients who underwent both EUS and surgery were enrolled: 15 patients whose tumors invaded the mucosa (m) or muscularis propria (mp) were classified as the “shallow group”, and 34 patients whose tumors invaded the ss were classified as the “deep group”. The EUS findings were compared between the two groups.

**Results:**

An irregular (narrow or thickened) LHEL was significantly more frequently observed on EUS in the deep group than in the shallow group. The diagnosis of ss invasion based on an irregular LHEL had the highest sensitivity and accuracy among the EUS imaging parameters (sensitivity 97.1% (33/34), specificity 86.7% (13/15), accuracy 93.8% (46/49)). When the deep group was limited to patients with a tumor depth of ss, the results were similar. When an irregular LHEL was used, the diagnostic accuracy of GBMTs with ss invasion was not significantly different between EUS specialists and beginners.

**Conclusions:**

The observation of an irregular (thickened or narrow) LHEL observed on EUS could be a reliable and simple method of diagnosing GBMTs with ss invasion and could contribute to choosing an appropriate surgical method.

## Background

Surgery is the only radical treatment for gallbladder malignant tumors (GBMTs). If the depth of tumor invasion is the mucosa (m) or muscularis propria (mp), cholecystectomy is sufficient. However, if the depth of tumor invasion reaches the subserosa (ss), approximately 50% of patients develop lymph node metastases [1–8]. In such cases, cholecystectomy is insufficient. Therefore, a preoperative diagnosis of GBMTs with ss invasion is important for the selection of the surgical method.

There are several modalities that can be used to closely investigate GBMTs. Transabdominal ultrasonography (US) and computed tomography (CT) are used first. However, the detectability on US is influenced by the physical status of the patients (e.g., subcutaneous fat), and it is difficult to observe gallbladder (GB) lesions in detail on CT. On the other hand, pancreatobiliary lesions can be visualized clearly from the inside of the upper gastrointestinal tract on endoscopic ultrasonography (EUS). Therefore, EUS is preferred over US or CT for the diagnosis of pancreaticobiliary diseases [9, 10]. In fact, many reports on the efficacy of EUS for diagnosing GBMTs have been published [11–23].

Compared to studies that aimed to improve GB cancer diagnosability by EUS, there have been few studies that reported on the diagnosability of the depth of GB invasion by EUS [17, 24–28]. In past reports, tumor depth was predicted by the shape of the wall of the GB on EUS imaging. The GB wall is depicted as a two-layer structure on EUS. These layers are the internal hypoechoic layer and lateral hyperechoic layer (LHEL). The internal hypoechoic layer involves the mucosal muscular layers and the superficial layer of the ss. The LHEL involves the serosal and ss layers [29, 30]. However, the classification of tumor depth based on the combination of tumor shape and the characteristics of the GB wall is slightly complicated, and it is difficult to judge whether the GBMT has invaded the superficial layer of the ss. Some GBMTs that have invaded the superficial layer of the ss do not show a narrowing of the LHEL. Therefore, in this study, we aimed to develop a simple and comprehensible method of diagnosing whether a GBMT has invaded the ss using EUS.

## Methods

### Study design and ethics

This study was a retrospective study that was performed to develop a simple method for diagnosing ss invasion by GBMTs. This study was approved by the Institutional Review Board of Fukushima Medical University.

### Patients

Forty-nine GBMT patients who underwent both EUS and surgery between May 2005 and September 2019 at Fukushima Medical University were enrolled in this study. Among them, 15 patients were ultimately diagnosed with a tumor invading the m or mp (m: nine patients, mp: six patients); these patients were classified as the “shallow group”. The remaining 34 patients were ultimately diagnosed with a tumor invading the ss; these patients were classified as the “deep group”. The final diagnosis was confirmed by investigation of surgical specimens.

### Preoperative diagnosis of GBMTs

GBMT patients first underwent US or contrast-enhanced CT (CECT). Then, the patients underwent EUS. After sedation with a transvenous administration of midazolam, an echoendoscope was gently inserted into the patients. GBMTs were observed through the antrum of the stomach or the duodenal bulbus.

The echoendoscopes used in this study were GF-UMP 230, GF-UM2000, GF-UC240P-AL, GF-UCT240-AL5, GF-UE260-AL5, and GF-UCT260 (Olympus Tokyo, Japan). The EUS systems used in this study were EU-M2000, EU-M30, EU-ME1 and EU-ME2 (Olympus Tokyo, Japan).

All EUS observations were performed by specialists who had performed more than 1000 pancreaticobiliary EUS procedures. No adverse events related to EUS were observed.

### Examination parameters

**Fig. 1:**
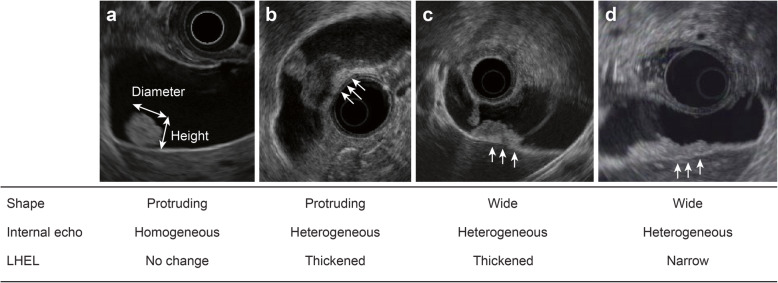
Definitions of EUS findings. **a**, The form of a homogeneous tumor was protruded. No change was observed in the LHEL. **b**, The form of a heterogeneous tumor was protruded. The LHEL became thick (arrows). **c**, The form of a heterogeneous tumor was wide. The LHEL became thick (arrows). **d**, The form of a heterogeneous tumor was wide. The LHEL became narrow (arrows). *LHEL*, lateral hyperechoic layer

Patient characteristics (age, sex), serum tumor markers (CEA, CA19–9), imaging findings, and histopathological findings (tumor type) were compared between the shallow and deep groups. The following imaging findings were assessed: tumor enhancement on CECT, maximum tumor diameter or height (measured on EUS), form (protruded or wide), internal echo (heterogeneous or homogeneous), and the LHEL observed with EUS. An irregular LHEL was defined as a thickened or narrow LHEL observed on EUS (Fig. 1). When a narrow LHEL is observed, ss invasion is obvious [24, 25, 27]; we also included a thickened LHEL in the examination parameters to achieve better diagnostic accuracy for ss invasion. CECT and EUS images were retrospectively reviewed by the consensus of more than two specialists who were blinded to the histologic depth of the GBMT. These specialists analyzed the image findings by discussing them with each other. Tumor type was determined as follows: Tubular, papillary, poorly differentiated adenocarcinoma were distinguished by tissue construction. A tumor that showed glandular structure was diagnosed as tubular adenocarcinoma. A tumor that showed papillary structure was diagnosed as papillary adenocarcinoma. An adenocarcinoma that lacked a glandular structure was diagnosed as poorly differentiated adenocarcinoma. A lesion that consisted of flat tumor cells was diagnosed as squamous cell carcinoma. Carcinosarcoma was diagnosed by observing pleomorphic tumor cells and performing epithelial immunostaining (AE1/3 (Roche Diagnostics, Tokyo, Japan), CK7 (Leica Biosystems, Nussloch, Germany)) and non-epithelial immunostaining (vimentin (Leica Biosystems), desmin (Leica Biosystems), S-100 (Roche Diagnostics), and SMA (DAKO, Glostrup, Denmark)). Neuroendocrine tumors were diagnosed by observing solid structure that consisted of small size cells and performing immunostaining (synaptophysin (Leica Biosystems), chromogranin A (Roche Diagnostics), and CD56 (ZYMED, Carlsbad, CA, USA)). Tumors with components of both neuroendocrine carcinoma and adenocarcinoma (30% or more of each) were diagnosed as adenoendocrine carcinoma.

After the most reliable method to diagnose ss invasion was determined, the usability of the method was compared between three pancreaticobiliary EUS beginners. The three less experienced EUS clinicians were endoscopists who did not fit the definition of a specialist. Pancreaticobiliary EUS specialists were defined as described above. EUS beginners diagnosed GBMTs with ss invasion by using the most efficient method.

### Statistical analyses

Continuous variables that did not follow a normal distribution were analyzed with the Mann-Whitney U test. Nominal variables were analyzed with Fisher’s exact test. *P* <  0.05 was defined as statistically significant. Bonferroni correction and Holm correction were used for multiple comparisons. All statistical analyses were performed using EzR (Saitama Medical Centre, Jichi Medical University, Saitama, Japan).

## Results

Age, sex, serum tumor marker levels, and tumor size were not significantly different between the shallow and deep groups (Table 1). Among the imaging findings, enhancement on CECT and internal echo (heterogeneous or homogeneous) were not significantly different between the shallow and deep groups. Wide-shaped tumors, an irregular LHEL, a thickened LHEL, and a narrow LHEL were significantly more common in the deep group than in the shallow group (Table 2). Tumor type was not significantly different between the shallow and deep groups. When patients in the deep group were limited to those with a tumor depth of the ss, these four parameters were significantly more frequently observed in the deep group (only the ss) than in the shallow group (Table 3).

**Table 1:** Comparison of patient characteristics and tumor size

	Shallow group (*N* = 15)	Deep group (*N* = 34)	*P* value
Age, years	71.0 (55–87)	78.5 (51–87)	0.071
Sex, male/female	6/9	16/18	0.76
Serum CEA, ng/ml	2.6 (1.1–8.0)	2.6 (0.8–889)	0.38
Serum CA19–9, U/ml	22.1 (2.8–139.4)	9.35 (0.1–864.9)	0.24
Tumor size, mm			
Maximum tumor diameter, mm	20.0 (4.5–40.0)	22.0 (10.0–80.0)	0.54
Tumor height, mm	15.0 (3.0–40.0)	15.0 (5.0–50.0)	1.0

Values are shown as the median (range) or n

**Table 2:** Comparison of imaging and histopathological findings

	Shallow group (*N* = 15)	Deep group (*N* = 34)	*P* value
Imaging findings			
Enhancement in CECT	14^a^ (100)	31^a^ (96.9)	1.0
EUS findings			
Form, protruded/wide	8/7	5/29	0.011
Internal echo, heterogeneous/homogeneous	14/1	34/0	0.31
Irregular (thickened or narrow) LHEL	2 (13.3)	33 (97.1)	< 0.01
Thickened LHEL	0 (0)	18 (52.9)	< 0.01
Narrow LHEL	2 (13.3)	28 (82.3)	< 0.01
Histopathological findings			
Tumor type			0.06
Tubular adenocarcinoma	5	22	
Papillary adenocarcinoma	9	7	
Poorly differentiated adenocarcinoma	1	1	
Carcinosarcoma	0	2	
Squamous cell carcinoma	0	1	
Adenoendocrine carcinoma	0	1	
Tumor depth			
m	9		
mp	6		
Deeper than the ss		34	

Values are shown as the median (range) or n (%) or n

^a^Some patients lacked CECT images

*CECT* Contrast-enhanced CT, *EUS* Endoscopic ultrasonography, *LHEL* Lateral hyperechoic layer, *m* Mucosa, *mp* Muscularis propria, *ss* Subserosa

**Table 3:** Comparison of EUS findings in patients whose tumors invaded the m-ss

	Shallow group (*N* = 15)	Deep group (only the ss) (*N* = 19)	*P* value
EUS findings			
Form, protruded/wide	8/7	3/16	0.03
Irregular (thickened or narrow) LHEL	2 (13.3)	18 (94.7)	< 0.01
Thickened LHEL	0 (0)	8 (42.1)	< 0.01
Narrow LHEL	2 (13.3)	15 (78.9)	< 0.01

Values are shown as n (%) or n

*m* Mucosa, *ss* Subserosa, *EUS* Endoscopic ultrasonography, *LHEL* Lateral hyperechoic layer

**Fig. 2:**
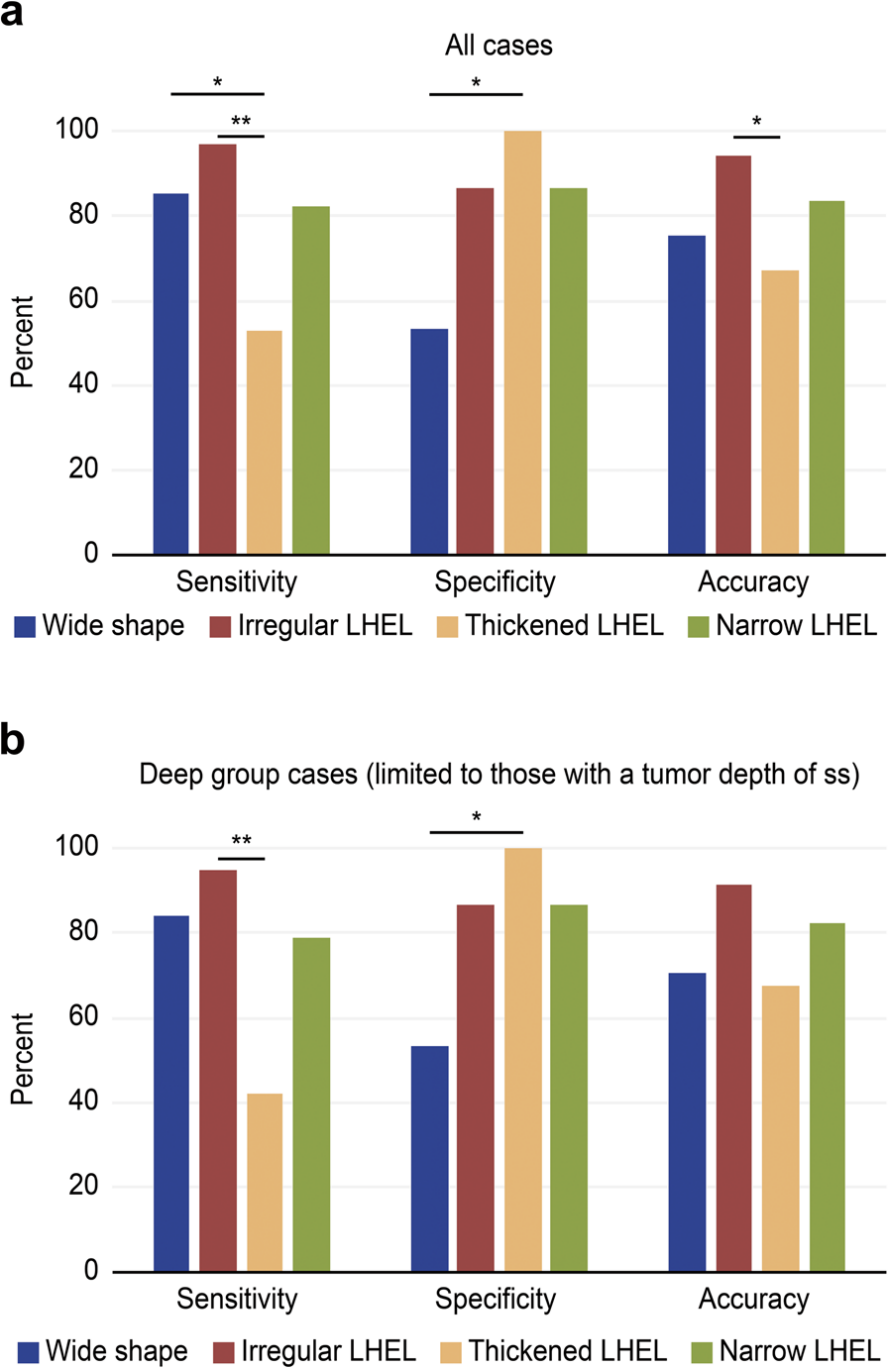
**a**, Comparison of the diagnosability of GBMTs with ss invasion by EUS imaging. An irregular LHEL showed the highest sensitivity and accuracy for diagnosing ss invasion among the four EUS imaging parameters. A thickened LHEL showed the highest specificity for diagnosing ss invasion among the four EUS imaging parameters. However, a thickened LHEL was less accurate than an irregular LHEL for diagnosing ss invasion. **b**, Diagnosability of GBMTs with ss invasion (limited only to the ss) on EUS. An irregular LHEL showed the highest sensitivity and accuracy for diagnosing ss invasion among the four EUS imaging parameters. *GBMT*, gallbladder malignant tumor, subserosa; *EUS*, endoscopic ultrasonography; *LHEL*, lateral hyperechoic layer. * *p* < 0.05, ** *p* < 0.01

Among the four EUS imaging parameters that were significantly different between the deep and shallow groups, the sensitivity and accuracy for diagnosing ss invasion of an irregular LHEL was the highest (sensitivity: 85.3% for wide shape (29/34) vs 97.1% for irregular LHEL (33/34) vs 52.9% for thickened LHEL (18/34) vs 82.4% for narrow LHEL (28/34), *P* < 0.01; accuracy: 75.5% for wide shape (37/49) vs 93.9% for irregular LHEL (46/49) vs 67.3% for thickened LHEL (33/49) vs 83.7% for narrow LHEL (41/49), *P* < 0.01) (Fig. 2a). Regarding the specificity for diagnosing ss invasion, a thickened LHEL was the most specific among the four EUS imaging parameters (53.3% for wide shape (8/15) vs 86.7% for irregular LHEL (13/15) vs 100% for thickened LHEL (15/15) vs 86.7% for narrow LHEL (13/15), *P* < 0.01). However, the sensitivity and accuracy for diagnosing ss invasion of a thickened LHEL was the lowest among the four EUS imaging parameters. When patients in the deep group were limited to those with a tumor depth reaching the ss, the sensitivity, specificity, and accuracy were similar (sensitivity: 84.2% for wide shape (16/19) vs 94.7% for irregular LHEL (18/19) vs 42.1% for thickened LHEL (8/19) vs 78.9% for narrow LHEL (15/19), *P* < 0.01; accuracy: 70.6% for shape of the tumor (24/34) vs 91.2% for irregular LHEL (31/34) vs 67.6% for thickened LHEL (23/34) vs 82.4% for narrow LHEL (28/34), *P* = 0.06). (Fig. 2b).

**Fig. 3:**
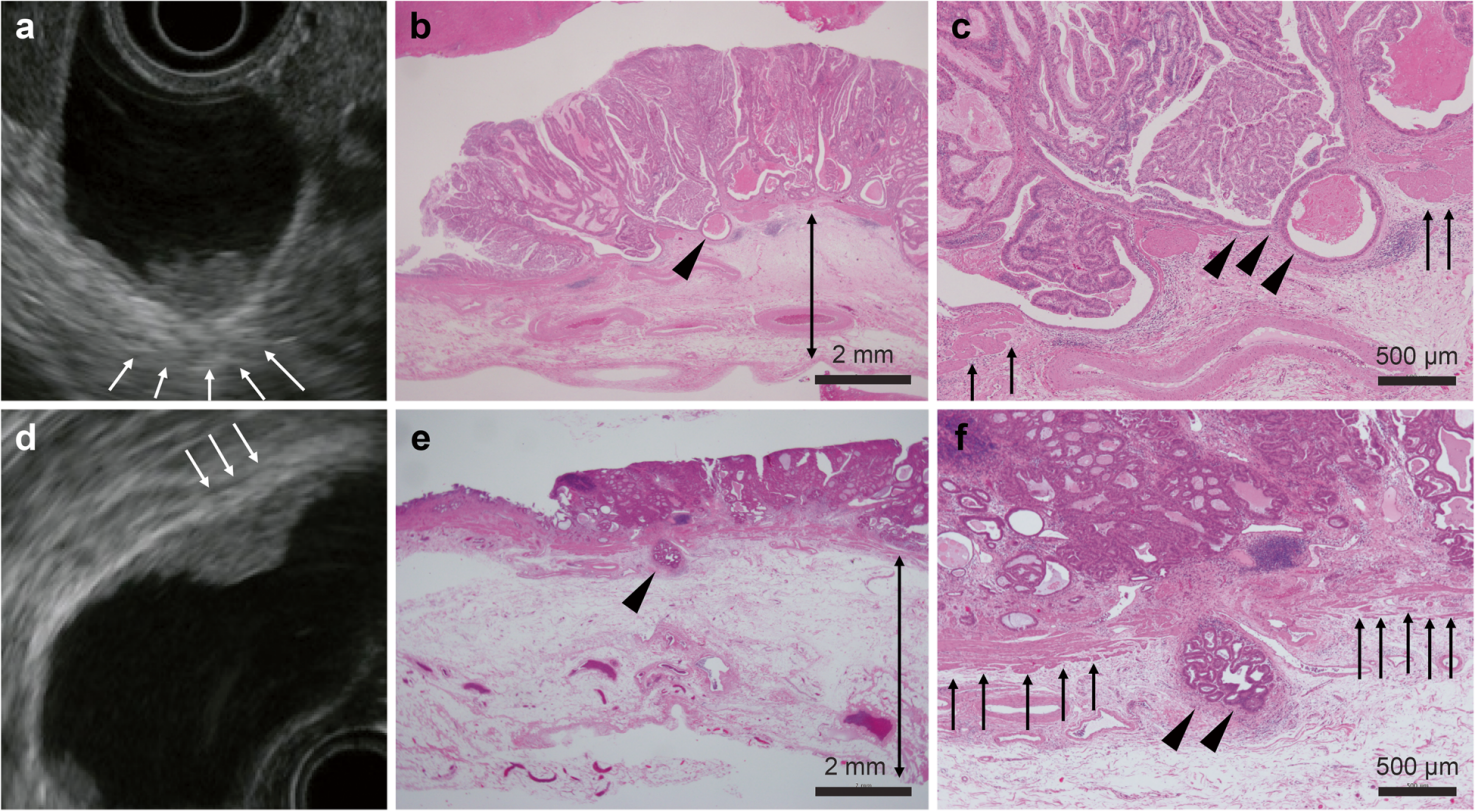
Histological findings of GBMTs with ss invasion. **a**, A GBMT was observed by EUS. The LHEL was thickened (arrows). **b**, Histological image of the specimen. The ss seemed edematous and was thickened (bar). **c**, Slight ss invasion was observed (arrowhead). Tumor invasion at the mp can be observed at both ends, with the tumor dividing the mp (arrows). **d**, A thickened LHEL was also observed in this specimen. **e**, The ss became thickened, as shown in Fig. 4b (bar). A GBMT with ss invasion was observed (arrowhead). **f**, The GBMT invaded the ss across the mp (arrowheads: ss invasion, arrows: mp). *ss*, subserosa; *GBMT,* gallbladder malignant tumor; *EUS*, endoscopic ultrasonography; *LHEL,* lateral hyperechoic layer; *mp*, muscularis propria

In this study, a thickened LHEL was observed only in the deep group. In patients whose tumors had slightly invaded the ss, edematous thickness of the ss was histologically observed (Fig. 3).

**Fig. 4:**
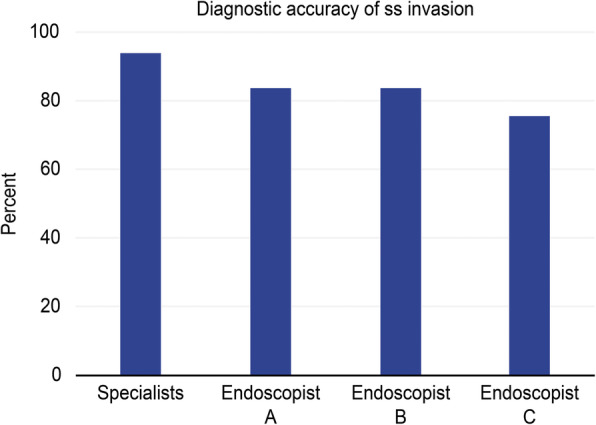
Comparison of the diagnostic accuracy of GBMTs with ss invasion between pancreaticobiliary EUS specialists and beginners. An irregular (thickened or narrow) LHEL was used to assess the diagnosability of GBMTs with ss invasion by three pancreaticobiliary EUS beginners (endoscopists **a**, **b**, and **c**) and specialists. The diagnostic accuracy of GBMTs with ss invasion was not significantly different between the specialists and beginners (specialists 93.9% vs beginners (Endoscopist A 83.7%, Endoscopist B 83.7%, and Endoscopist C 75.5%)). *GBMT,* gallbladder malignant tumor; *ss*, subserosa; *EUS,* endoscopic ultrasonography; *LHEL,* lateral hyperechoic layer

An irregular LHEL was then used as the diagnostic parameter for the ss invasion of GBMTs, and the diagnostic accuracy was compared between pancreaticobiliary EUS specialists and beginners. The diagnostic accuracy of ss invasion was not significantly different between the specialists and three beginners (endoscopists A, B, and C) (Fig. 4).

## Discussion

In this study, we developed a simple method that can be used to diagnose the ss invasion of GBMTs on EUS. In past reports, a narrow LHEL was recognized as a sign of ss invasion. In this study, a thickened LHEL was also indicative of ss invasion. As a result, an irregular (thickened or narrow) LHEL was determined to be a simple and reliable EUS finding that could be used to diagnose GBMTs with ss invasion. An irregular LHEL was also reliable if the patients were limited to those with a tumor depth of m-ss. By using this method, pancreaticobiliary EUS beginners can diagnose GBMTs with ss invasion.

As described in the beginning of this paper, the depth of GB invasion was determined by the combination of tumor form and the structure of the GB wall under the tumor. Regarding the form, the depth of pedunculated tumor invasion is usually the m. In a case of this study, a definite stalk was observed by EUS, with a tumor depth of m. However, when wide-shaped lesions are involved, any depth of invasion can be observed [25, 27]. In this study, the specificity for diagnosing ss invasion was low when the form of the tumor was used. Therefore, the form of the tumor was removed from the simple method developed to diagnose ss invasion, while EUS findings of the GB wall were retained.

Some decades ago, it was believed that the internal hypoechoic layer involved the mucosal muscular layers and that the LHEL involved the serosal and subserosal layers [24]. Currently, it is known that the superficial layer of the ss constitutes a part of the internal hypoechoic layer [29, 30]. As described in the introduction, there have been few reports on the efficacy of EUS for diagnosing the depth of GBMT invasion. In these reports, an LHEL that was thinned or disrupted by the tumor indicated tumor invasion deeper than the ss [24, 25, 27]. However, a problem existed in these reports: ss invasion in patients in whom the LHEL was not narrow or disrupted was not examined. In this report, a thickened LHEL was included as a type of irregular LHEL. Our ability to diagnose the ss infiltration of GBMTs was superior to that described in previous reports. In fact, when only a thickened LHEL was observed, the ss that was slightly invaded by the GBMT was observed to be edematous and thickened in pathological specimens (Fig. 3).

It is difficult to diagnose GBMT with invasion into the ss without a narrow or disrupted LHEL, although several attempts have been made. In 2002, Kimura et al. [26] performed EUS and angiography to diagnose ss invasion. When angiography was performed on patients to assess the LHEL, those with no abnormal findings at the cystic artery or its branches were diagnosed as having m or mp GBMTs. On the other hand, patients with abnormal findings at the second or third branches of the cystic artery were diagnosed as having GBMTs with ss invasion. Good diagnosability of GBMTs with ss invasion was reported when these methods were used (sensitivity 81.8%, specificity 90.6%, accuracy 88.4%) [26]. In 2019, Sakamoto et al. [28] reported a scoring system that could be used to diagnose GBMTs with ss invasion: − 3.954 + 0.555 CEA + 0.094 x diameter of the GBMT. The sensitivity and specificity using this score for diagnosing ss invasion were 85.0 and 87.1%, respectively (cut-off value − 0.584). Although these methods are efficient for diagnosing GBMTs with ss invasion, angiography is invasive, and the diameter of a wide GBMT is difficult to measure. Therefore, a simple and widely applicable method is desirable. In this report, we developed a simple method using EUS findings of a narrow or thickened LHEL.

This study has some limitations. First, the study design is retrospective, and it was performed at a single institution. Second, EUS is influenced by the technique of the endoscopist. However, EUS was performed by pancreaticobiliary specialists in this study. Therefore, the quality of the EUS image should have been maintained. Third, the evaluation of EUS images is subjective to a certain degree. To overcome this limitation, the diagnostic method used in this study was also evaluated by pancreaticobiliary EUS beginners. In fact, the efficacy of this method was retained in the beginners. Fourth, edematous thickening of the GB wall can be caused by inflammation, and this inflammation can affect the diagnosis of shallow group. In this study, no definite clinical symptoms or elevated serum CRP suggestive of inflammation was observed in the shallow group (CRP: 0.03–0.74 mg/dl). Serum CRP was measured at the time EUS was scheduled or within a week before or after EUS. When the EUS diagnostic method in this study is used, the existence of cholecystitis should be confirmed. Fifth, the EUS methods of this study cannot diagnose a GBMT with certain types of cholecystitis, such as hyalinizing cholecystitis (HC), which causes damage to the layer structure of the GB wall [31–33]. HC is strongly associated with GB cancer. In addition, the diagnosis of HC-related cancer is difficult. Because HC-related cancer does not show a definite mass, when the layer structure of GB wall cannot be identified by EUS, the possibility of HC should be considered.

## Conclusions

EUS findings of a narrow or thickened LHEL were observed mainly in patients with GBMTs with ss invasion. This reliable and simple method can be used to diagnose GBMT invasion that is deeper than the ss and may contribute to the appropriate selection of the operative method.

## Data Availability

The datasets generated and/or analyzed during the current study are available from the corresponding author upon reasonable request.

## Abbreviations

GBMT: Gallbladder malignant tumor
ss: Subserosa
EUS: Endoscopic ultrasonography
LHEL: Lateral high echoic layer
m: Mucosa
mp: Muscularis propria
US: Ultrasonography
CT: Computed tomography
GB: Gallbladder
CECT: Contrast-enhanced CT
